# Articles

**Published:** 1839-08

**Authors:** E. Noyes


BALTIMORE, June 12th, 1839.

DRS. HARRIS & PARMLY,

Gentlemen,—

I have the pleasure of acknowledging the receipt of the first number of "THE AMERICAN JOURNAL OF DENTAL SCIENCE," the appearance of which I hail as the harbinger of a more auspicious condition of our art. The importance of a publication of this kind, not only to our own immediate profession, but also to that of medicine generally, must be apparent to every reflecting mind: it has long been needed, and will, I little doubt, receive a cordial and liberal support. That it will be sustained, I fell confident; it would be a reproach to the profession, for whose more especial benefit it is designed, were it not to receive a support sufficient to justify its continuance. For my own part, I am so much pleased with it that I have ordered twenty copies, and would subscribe for more, rather than the publication should cease.

The literature of our art, or at least that which is available to the great body of the profession, is at most meagre; and now that an opportunity is offered whereby every one may furnish himself, and that too at a small price, with all that is valuable of that which now exists, I should suppose that every one having any pretensions to science, would gladly embrace it. In addition to this, the present stock will be continually enriched by new contributions, and thus all will be enabled to keep pace with the improvements that are daily being made in the theory and practice of Dentistry, which of itself, ought to be a sufficient inducement to every one of its practitioners to become a patron of the present publication.

It is unfortunate for our profession that admission to its membership is but too often gained by persons possessing none of the requisite qualifications, and that among this class of practitioners, a disposition is manifested, with a view to conceal their ignorance, to veil the art with as much mystery as possible. But to a proper appreciation of its benefits, a more general and extensive knowledge of its principles is necessary; for the dissemination of which, and for the exposure of the impositions daily practised by such persons, a periodical publication, devoted to the science of dentistry, is eminently calculated.

Among the subjects proposed to be treated in the *Journal*, I observe in the prospectus of the *Publishing Committee*, it is stated that "the arts of quackery will be boldly exposed." This is as it should be. The profession has already suffered too much from the empyricism of artful knavery and the mal-practice of the uninformed. Of the former, the *notorious* CRAWCOURS, who visited the United States three or four years since are a striking example. They, with their "ROYAL MINERAL SUCCEDANEUM," taught many of the people in this country a lesson which one would suppose would have guarded them against any future impositions of the kind. But is this the fact; are they any more cautious of those who pursue a similar practice? No. The rooms of those who advertise to plug teeth with a *mineral*, or *metalic cement*, or *paste*, as it is sometimes called, are generally thronged with applicants for the almost magical services of their occupants. No matter how ignorant a man may be of the principles of the art if he only has effrontery enough to come out with a blazing advertisement and propose to restore all kinds of diseased teeth to health and usefulness, however far gone they may be, and to supply the places of lost ones by a species of legerdemain, he is sure to have plenty of business. Were the persons who seek the services of this description of impostors really ignorant of the pernicious effects of their practice, it would not be a matter of much surprise; but that well informed individuals should suffer themselves to be thus easily duped, is indeed truly astonishing.

The plugging of teeth with *metalic cement*, *pastes*, *&c.* or more properly speaking, with *amalgums* of *mercury* and *tin*, *silver* or *zinc*, though the quacks would not dare to proclaim this to the world, as it would then blast their prospects of future success in such infamous CRAWCOURISM, is a species of quackery that ought to be suppressed. Of the pernicious effects of this practice, the public ought to be made acquainted; and I for one would be highly gratified to see some remarks upon the subject by you or some other person capable of doing it justice.

Respectfully,
I have the honor to be, sirs,
Your ob't serv't,
E. NOYES.

